# Evolving Strategies in Pediatric Cataract Surgery: A 7-Year Analysis of Ocular Biometry and IOL Localization

**DOI:** 10.3390/jcm15166202

**Published:** 2026-08-11

**Authors:** Luca Schwarzenbacher, Belma Strugalioska, Gregor S. Reiter, Lorenz Wassermann, Sandra Rezar-Dreindl, Stefan Sacu, Eva Stifter

**Affiliations:** 1 Department of Ophthalmology and Optometry, Medical University of Vienna, Währinger Gürtel 18-20, 1090 Vienna, Austriastefan.sacu@meduniwien.ac.at (S.S.);; 2 Laboratory for Ophthalmic Image Analysis, Medical University of Vienna, Spitalgasse 23, 1090 Vienna, Austria

**Keywords:** pediatric cataract, temporal trends, intraocular lens localization, ocular biometry, surgical planning

## Abstract

**Objectives:** To evaluate temporal trends in ocular biometrics, patient demographics and surgical strategies for intraocular lens (IOL) localization in pediatric cataracts. **Methods:** This retrospective study included 291 eyes of 148 pediatric patients operated at a tertiary referral center between 2019 and 2025, comprising 219 operated eyes and 72 unaffected fellow eyes serving as intra-individual controls. Eyes were stratified into three chronological cohorts (2019–2020, 2021–2022, 2023–2025). Axial length (AL), white-to-white (WTW) diameter and central corneal thickness were analyzed in relation to the anatomical IOL placement. A generalized linear mixed model with a random intercept per patient accounted for the within-patient correlation between fellow eyes. **Results:** Mean AL increased from 18.91 ± 2.83 mm to 20.32 ± 3.19 mm (*p* = 0.004), whereas the increase in WTW diameter was not significant (*p* = 0.450). The age at surgery increased significantly across the periods (median 1.08, 1.33 and 3.13 years; *p* < 0.001), a general temporal effect that was not modified by the site of implantation (*p* = 0.32). Underlying etiology strongly influenced the approach: all eyes with Marfan syndrome received an iris-fixated IOL, whereas 90% of uveitic eyes remained aphakic. Femtosecond laser-assisted cataract surgery (FLACS) was used in 13 eyes and confined to the most recent period. **Conclusions:** Pediatric cataract surgery shows a progressive increase in the age at surgery that is not specific to any IOL localization, while the surgical approach is frequently determined by the underlying etiology. The increasing use of FLACS represents an institutional trend; the small number of cases and absence of outcome data preclude conclusions regarding its safety or feasibility.

## 1. Introduction

Over the past decades, pediatric cataract surgery has become an increasingly standardized, anatomy-based procedure aimed at maintaining a clear visual axis while minimizing long-term complications. Contemporary approaches typically combine lens aspiration with deliberately planned anterior and posterior capsulotomy concepts and in many cases adjunctive anterior vitrectomy, optic capture techniques and, when feasible, primary intraocular lens (IOL) implantation [1,2].

Evidence from large longitudinal cohorts indicates that visual axis opacification and glaucoma-spectrum disease remain the most relevant long-term complications after pediatric cataract surgery [3]. These findings highlight that the chosen surgical technique, particularly the management of the posterior capsule and the decision to perform anterior vitrectomy, can be as critical as the timing of the initial intervention itself [4]. Furthermore, the decision regarding the exact anatomical placement of the IOL—whether in the capsular bag, the ciliary sulcus, or via anterior chamber/iris fixation—is highly dependent on patient age and underlying etiologies, such as connective tissue disorders like Marfan syndrome [5,6].

In parallel, femtosecond laser-assisted cataract surgery (FLACS) has gradually transitioned from a technology primarily used in adult refractive cataract surgery to a potentially valuable adjunct in pediatric cases. One of the main advantages of femtosecond platforms is the ability to create highly reproducible capsulotomies, which represents one of the technically most demanding steps in pediatric surgery due to increased capsular elasticity and a higher risk of capsular tears. Early clinical series using low-energy femtosecond systems have demonstrated encouraging feasibility and safety profiles in selected pediatric cases, particularly where precise capsulotomy geometry is essential for stable in-the-bag or bag-in-the-lens IOL fixation [7,8].

Congenital and acquired pediatric lens opacities remain a major cause of preventable visual impairment in childhood and successful management continues to depend on accurate ocular biometry and careful surgical planning. Unlike adult eyes, the pediatric eye undergoes ongoing anatomical development after surgery, characterized by continued axial elongation and substantial biomechanical changes. These developmental processes influence refractive outcomes and the long-term stability of implanted intraocular lenses [9].

While our previous work emphasized the importance of baseline biometric parameters such as axial length and corneal diameter for perioperative decision-making, significantly less is known about how these parameters and the associated surgical strategies have evolved over time within clinical practice [10]. Understanding these temporal developments may provide valuable insights into changing patient characteristics, technological adoption and subtle, yet critical, shifts in surgical decision-making.

Against this background, the present follow-up study aims to examine temporal changes in ocular biometrics and surgical strategies within a pediatric cataract cohort treated at a tertiary referral center. Specifically, we investigate how patient characteristics, biometric parameters, intraocular lens implantation strategies have evolved over time as well as how surgical technologies and clinical practice patterns have developed.

The primary objective of this study is therefore to quantify temporal trends in ocular biometric parameters and surgical decision-making, including the use and positioning of primary IOL implantation across three treatment periods between 2019 and 2025. Additionally, we aim to elucidate how specific underlying conditions, such as traumatic cataracts and Marfan syndrome, continue to uniquely dictate individualized surgical approaches within this evolving landscape.

## 2. Materials and Methods

The study was approved by the Ethics Committee of the Medical University of Vienna (approval number 1116/2019) and was conducted in accordance with the Declaration of Helsinki. Due to the retrospective design of the study, the requirement for informed consent was waived.

In this retrospective follow-up study, we reviewed the medical records of all consecutive pediatric patients (aged under 18 years) who underwent cataract extraction for unilateral or bilateral cataracts at the Medical University of Vienna between 2019 and 2025. Cataracts were classified by etiology as congenital, developmental or infantile, traumatic or uveitic. Only primary procedures were analyzed; secondary intraocular lens implantations and repeat surgeries were not counted as separate cases and eyes with prior intraocular surgery unrelated to cataract were excluded. Eyes with missing biometric values were retained without imputation and the number of eyes contributing to each parameter is reported accordingly.

To evaluate temporal changes in ocular biometric characteristics and surgical management, the study cohort was divided into three equally sized groups according to the time of surgery (2019–2021, 2022–2023 and 2024–2025).

IOLs were implanted either in the capsular bag, in the sulcus or iris-fixated lenses, whereas some eyes were left aphakic depending on the individual anatomical conditions and specific clinical requirements; the site of fixation was determined intraoperatively according to the available capsular and zonular support. Femtosecond laser-assisted cataract surgery (Ziemer LDV Z8, Port, Switzerland) was performed when the white-to-white diameter exceeded 11.5 mm and the pupil diameter exceeded 6 mm, in the absence of corneal pathology. Furthermore, to assess the specific impact of IOL standardization on surgical decision-making, we introduced a sub-analysis comparing anatomical IOL localizations before and after March 2023, when a standardized 3-piece IOL platform (Avansee Preset IOL, Kowa Company, Ltd., Nagoya, Japan) became the primary choice at our institution. In addition, unaffected fellow eyes of patients with unilateral cataract were included as controls when applicable and served as an intra-individual biometric reference, without entering the comparisons of surgical strategy.

### 2.1. Examinations

All patients underwent a standardized preoperative ophthalmic examination under general anesthesia immediately prior to cataract surgery. In unilateral cases, the unaffected fellow eye was also examined and included as an intraindividual control where applicable. The specific examination protocol utilized for the current analysis comprised intraocular pressure measurement by indentation tonometry (Schiötz, Rudolf Riester GmbH, Jungingen, Germany; averaging the 5.5 g and 10.0 g weight values), white-to-white (WTW) corneal diameter measurement using a surgical caliper, central corneal thickness (CCT) assessment with a pachymeter (Quantel Medical Pocket II, Cournon-d’Auvergne, France), axial length (AL) measurement by ultrasound A-scan (Quantel Medical Aviso, Cournon-d’Auvergne, France) and flat keratometry (Kf) and steep keratometry (Ks) were measured with a hand-held refractometer (Retinomax 5, Bon Optic, Lübeck, Germany).

All measurements were obtained in a predefined standardized order to avoid interference between examination steps or artificially altered intraocular pressure readings. In addition to these biometric and tonometric data, patient demographics (including age at surgery and sex) and specific underlying etiologies—namely Marfan syndrome, ocular trauma and uveitis—were systematically recorded.

This standardized approach ensured highly reliable baseline assessments and allowed for a precise comparison of preoperative ocular characteristics across the three chronologically defined study periods.

### 2.2. Statistical Analysis

The statistical evaluation of our dataset was conducted with the aim of assessing temporal trends across three consecutive study periods and evaluating differences in age and preoperative ocular biometrics (axial length, white-to-white diameter and central corneal thickness) based on IOL localization strategies.

Preliminary analyses included descriptive statistics to summarize the central tendency and dispersion of the data. Normality of the continuous variables was assessed using the Shapiro–Wilk test and quantile–quantile plots. Age at surgery deviated significantly from a normal distribution in all three study periods (W = 0.768, 0.746 and 0.851; df = 99, 90 and 102; all *p* < 0.001) and the quantile–quantile plots showed corresponding departures from the reference line, particularly in the tails. Continuous variables were therefore reported as the median together with minimum and maximum values, except for subgroups comprising fewer than ten eyes, in which the mean ± standard deviation (SD) is given because quantile-based summaries are unstable at these sample sizes. Categorical variables were presented as absolute frequencies and percentages.

Given this non-normal distribution, non-parametric methods were used for the descriptive comparisons. The Kruskal–Wallis test was applied to compare continuous biometric variables across the three chronological study periods (2019–2020, 2021–2022 and 2023–2025).

Because many patients contributed both eyes and biometric measurements of fellow eyes are correlated, the assumption of independence underlying these tests was addressed with a generalized linear mixed model, which provided the primary inference of the study. Age at surgery was modeled with study period, IOL localization and their interaction as fixed effects and a random intercept for each patient, thereby accounting for the within-patient correlation between fellow eyes. Between-period differences were derived as estimated marginal means and compared using sequentially Bonferroni-adjusted pairwise contrasts. The Bonferroni correction was applied specifically to these post hoc pairwise contrasts and not to the descriptive statistics or the omnibus test.

All statistical tests were two-tailed and a *p*-value of less than 0.05 was considered to indicate statistical significance. The analyses were performed using IBM SPSS Statistics (version 29.0, IBM Corp., Armonk, NY, USA).

## 3. Results

The baseline demographics and ocular parameters of the study population, categorized by the chronological study period (Group 1: 2019–2020; Group 2: 2021–2022; Group 3: 2023–2025), are summarized in Table 1. The cohort comprised a total of 291 eyes from 148 patients. Group 1 included 99 eyes (50 patients), Group 2 included 90 eyes (47 patients) and Group 3 included 102 eyes (51 patients).

The median age at surgery increased across the three periods, from 3.32 years in Group 1 to 3.29 years in Group 2 and 4.68 years in Group 3; this increase was confirmed as statistically significant by the mixed-effects model described below (*p* < 0.001). The most frequently identified underlying conditions across all groups included traumatic cataract, Marfan syndrome and uveitis, although the incidence of these specific etiologies remained consistently low (≤5.1% per group, Table 1).

Regarding preoperative ocular biometrics, an increasing trend over time was observed for axial length, advancing from 18.91 ± 2.83 mm in Group 1 to 20.32 ± 3.19 mm in Group 3 (*p* = 0.004). Mean white-to-white corneal diameter showed a slight, non-significant increase from 11.19 ± 1.19 mm in Group 1 to 11.46 ± 1.16 mm in Group 3 (*p* = 0.450). Central corneal thickness varied across the cohorts, measuring 590.88 ± 53.96 μm in Group 1, 570.99 ± 46.16 μm in Group 2 and 597.60 ± 62.04 μm in Group 3 (*p* = 0.012). The sex distribution of the operated eyes showed a slight male predominance in Group 1 (51.5%) and a marked male predominance in Group 3 (73.5%), whereas Group 2 was predominantly female (62.2%).

**Figure 1:**
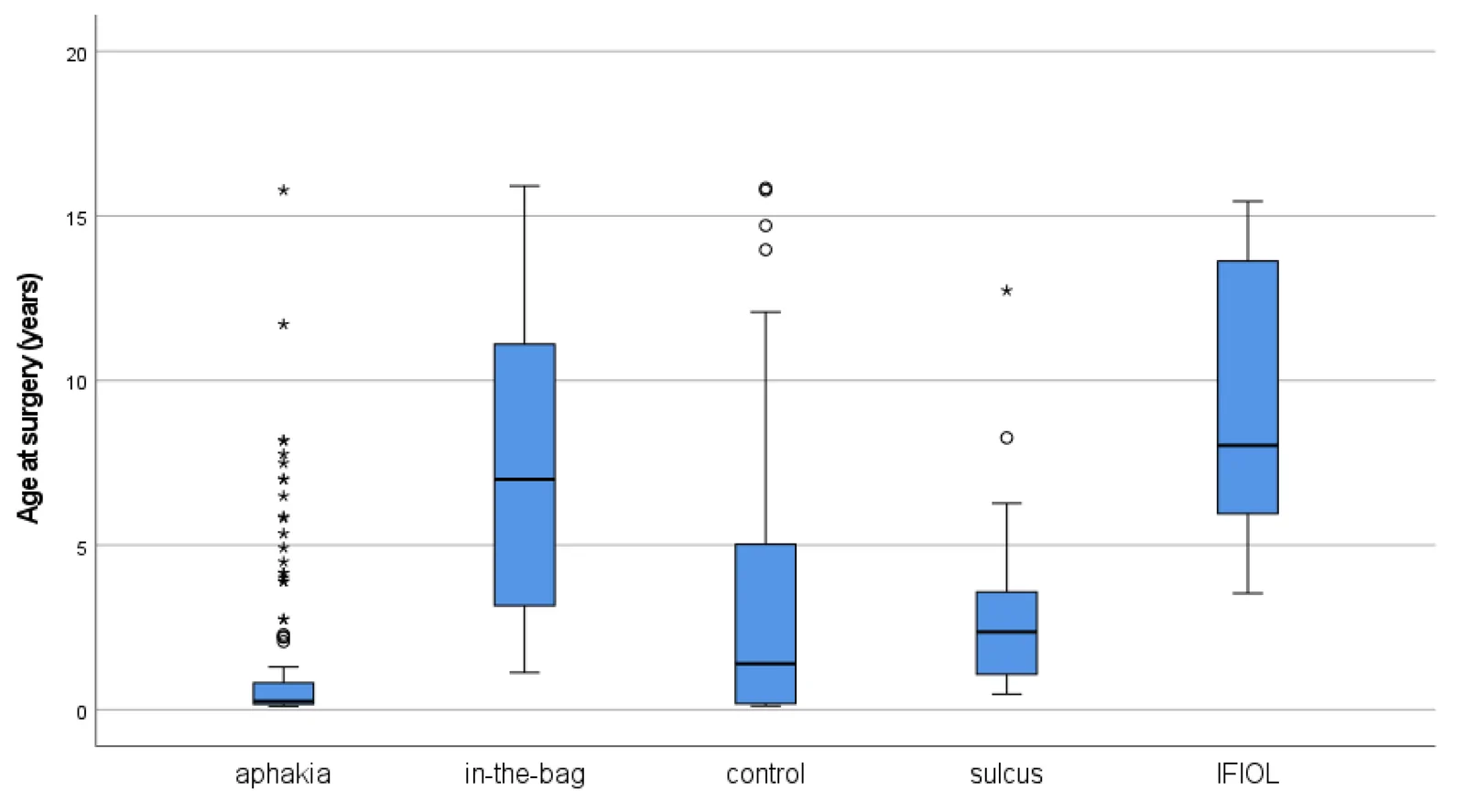
Distribution of age at surgery according to intraocular lens (IOL) localization. Box-and-whisker plots illustrating the age at surgery (in years) for the four surgical categories (aphakia, in-the-bag, ciliary sulcus and iris-fixated IOL [IFIOL]) together with the unaffected fellow eyes serving as controls. The central horizontal line within each box represents the median age, while the lower and upper hinges of the box indicate the first (25th) and third (75th) quartiles, respectively. The whiskers extend to the minimum and maximum values within 1.5 times the interquartile range (IQR). Circles represent outlier data points and asterisks (*) denote extreme outliers. The figure is descriptive; the statistical comparison of age across study periods was performed with the mixed-effects model reported in the Section 2 and Section 3.

The distribution of the age at surgery varied by surgical approach, as summarized in Table 2 and illustrated in Figure 1. Eyes managed with primary aphakia presented with the lowest median age at surgery across all periods (0.24, 0.26 and 0.38 years in Groups 1 to 3). The control eyes likewise showed a low median age throughout (1.38, 0.85 and 2.67 years).

In contrast, eyes receiving an in-the-bag lens were older, with median ages of 5.92, 7.69 and 6.70 years across the three periods and a correspondingly wide age range. The iris-fixated (IFIOL) subgroup was small (5, 1 and 2 eyes across the periods) and its age is therefore reported as the mean ± standard deviation, amounting to 11.76 ± 3.65 years in Group 1, a single eye aged 3.54 years in Group 2 and 5.96 ± 0.52 years in Group 3.

Descriptively, the age at surgery within the ciliary sulcus subgroup increased across the three study periods, from a mean of 1.49 ± 1.11 years in the first period to 3.03 ± 2.97 years in the second and 4.80 ± 3.48 years in the third (Table 2); a comparable increase was not evident in the other localization subgroups. This is a descriptive observation only. In the mixed-effects analysis, the increase in age at surgery was a general effect across the whole cohort (F(2, 276) = 89.5, *p* < 0.001) that was not significantly modified by the site of implantation, as the interaction between IOL localization and study period was not significant (F(8, 276) = 1.17, *p* = 0.32). Given the small number of sulcus cases (*n* = 21), this apparent trend should therefore be regarded as a descriptive finding rather than as evidence of a lens-specific evolution in surgical indication.

Table 3 comprises all 219 operated eyes and presents the complete spectrum of primary surgical decisions. Of these, 103 eyes received a primary intraocular lens (74 in-the-bag, 21 ciliary sulcus and 8 anterior chamber or iris-fixated) and 116 eyes were left primarily aphakic. Aphakia is therefore reported here as a distinct primary surgical decision and not as an intraocular lens localization; the localization sub-analysis referred to in the main text pertains to the 103 eyes that received a primary intraocular lens. Values represent frequencies (*n*) and column percentages (%). The distribution of surgical decisions did not differ significantly between the two eras (Pearson chi-square, χ^2^(3, *N* = 219) = 2.18, *p* = 0.54).

To evaluate the impact of standardizing the intraocular lens type on surgical decision-making, we analyzed the primary surgical decision, comprising the anatomical localization of the implanted lens and primary aphakia, in all 219 operated eyes before and after the introduction of a specific 3-piece IOL (Avansee Preset IOL, Kowa Company, Ltd., Nagoya, Japan) in March 2023. Of these 219 eyes, 103 received a primary intraocular lens and 116 were left primarily aphakic. Before March 2023 (153 operated eyes), during which various IOL models were used, 55.6% of eyes were left aphakic, 30.7% received an in-the-bag lens, 9.8% a ciliary sulcus lens and 3.9% an iris-fixated (IFIOL) lens. Following the transition to the standardized 3-piece platform (66 operated eyes), the proportion of aphakia decreased to 47.0% and in-the-bag placement increased to 40.9%, whereas ciliary sulcus fixation (9.1%) and iris-fixated placement (3.0%) remained largely unchanged. The overall distribution of surgical decisions did not differ significantly between the two eras (χ^2^(3, *N* = 219) = 2.18, *p* = 0.54).

Femtosecond laser-assisted surgery was used in 13 eyes, corresponding to 4.5% of the cohort, with a median age at surgery of 6.70 years (range 1.13–14.71 years). Its use was confined to the most recent study period: it was not performed in Group 1 or Group 2, whereas in Group 3 it was applied in 13 of 102 eyes (12.7%). This pattern reflects the recent introduction of the technique at our institution rather than a gradual increase across the whole study period.

## 4. Discussion

In the present study, we conducted a comprehensive longitudinal analysis of pediatric cataract patients, evaluating ocular biometric parameters and surgical strategies across three consecutive time periods between 2019 and 2025. By stratifying our cohort into chronological groups, we aimed to identify temporal trends in surgical decision-making, specifically regarding IOL localization and the age at intervention. Our findings provide valuable insights into the evolving optimization of surgical strategies for the treatment of congenital and pediatric cataracts.

Our data demonstrate a progressive increase in the age at surgery across the study period, which was confirmed in a correlation-adjusted mixed-effects analysis. Importantly, this increase was a general temporal trend affecting the cohort as a whole rather than an effect specific to any individual IOL localization. The relative ordering of the surgical strategies, however, remained consistent throughout, indicating well-established and standardized clinical algorithms at our center.

Consistent with our previous observations and current literature, in-the-bag IOL placements were predominantly reserved for older pediatric patients (median ages ranging from 5.9 to 7.7 years across periods). These older children typically present with larger corneal diameters and longer axial lengths, thereby offering superior capsular bag stability and structural support. Conversely, the aphakic approach remained the standard of care for the youngest cohort (median age below 6 months in all periods). This reflects the ongoing consensus that primary IOL implantation in infants under one to two years of age carries a significantly higher risk of severe visual axis opacification (VAO), secondary glaucoma and massive refractive shifts due to the rapid ocular growth phase [3].

When analyzing specific underlying etiologies within our cohort, namely Marfan syndrome, traumatic cataracts and uveitis-associated cataracts, distinct clinical trends regarding IOL localization emerge. All eyes with Marfan syndrome were treated with iris-fixated IOLs (IFIOL) at a notably older age (mean 9.7 years). This absolute preference reflects the inherent, severe lack of zonular integrity characteristic of Marfan syndrome, which typically renders standard capsular bag or ciliary sulcus implantation unfeasible or highly susceptible to late intraocular dislocation.

In contrast, eyes with traumatic cataracts exhibited a heterogeneous surgical approach highly dependent on the severity and extent of the anterior segment injury. While a portion of these cases successfully received a primary IOL (such as in-the-bag or sulcus implantations) if the capsular architecture was largely preserved, severe penetrating traumas frequently necessitated an initial aphakic strategy. This emphasizes that in the setting of pediatric ocular trauma, primary bag implantation is actively pursued to facilitate visual rehabilitation when structurally viable; however, extensive structural disruption reliably forces a delay in IOL placement, leading to primary aphakia.

Furthermore, the management of uveitis-associated cataracts in our pediatric cohort overwhelmingly favored an aphakic strategy (9 out of 10 eyes; mean age 3.5 years). Only a single patient received an in-the-bag IOL. This profound trend aligns with the prevailing clinical caution in pediatric uveitis, where primary IOL implantation is often avoided due to the heightened risk of exacerbating intraocular inflammation, fostering dense posterior synechiae and promoting severe visual axis opacification or cyclitic membranes [11]. Across all three of these specific etiologies, our data clearly illustrate that standard age-based biometric paradigms are frequently overruled by the unique anatomical and inflammatory constraints dictated by the underlying disease.

While our cohort predominantly received iris-fixated IOLs in cases of profound zonular insufficiency, it is important to acknowledge the recent evolution of surgical alternatives. Currently, sutureless flanged intrascleral haptic fixation, commonly known as the Yamane technique, is increasingly utilized as a preferred method for secondary IOL placement when capsular support is absent [12]. Although this specific technique was not represented in our dataset, its growing adoption in pediatric ophthalmology reflects its potential to preserve anterior segment anatomy and mitigate long-term complications, such as endothelial cell loss or secondary glaucoma, which are historically associated with anteriorly fixated lenses [13].

Descriptively, the age of patients receiving ciliary sulcus IOLs showed the most pronounced increase in all localization subgroups, rising from a mean of 1.49 ± 1.11 years in the first period to 3.03 ± 2.97 years in the second and 4.80 ± 3.48 years in the third. This apparent evolution would be consistent with a refined surgical caution. It must, however, be interpreted with restraint: the subgroup is small (*n* = 21) with a high standard deviation and in the correlation-adjusted mixed-effects analysis the temporal increase was not significantly modified by the site of implantation, indicating that this observation reflects the general temporal trend rather than a sulcus-specific change in indication. The rationale for such caution is nevertheless well founded, as the ciliary sulcus in very young eyes is underdeveloped and might not provide adequate long-term support for IOL haptics, potentially leading to decentration, uveal irritation, or secondary pigment dispersion [14,15]. Consequently, for young pediatric patients in whom in-the-bag implantation is precluded due to capsular compromise or anatomical constraints, delaying sulcus implantation or opting for extended aphakia with contact lens correction appears to be an increasingly favored approach.

In our cohort, the introduction of a standardized 3-piece IOL platform in March 2023 was a further pivotal step in addressing the unpredictable nature of pediatric cataract surgery. While this transition did not significantly alter the primary anatomical localization of the lenses, it proved clinically beneficial by ensuring a versatile platform. This architecture provides robust capsular support while inherently maintaining the flexibility required for safe ciliary sulcus fixation or secondary salvage interventions, such as the Yamane technique, should capsular integrity be compromised.

Regarding ocular biometrics, we observed a progressive increase in mean axial length from 18.91 mm to 20.32 mm across the study periods, whereas the increase in white-to-white (WTW) corneal diameter from 11.19 mm to 11.46 mm did not reach statistical significance. This trend is likely a direct reflection of the slightly older overall patient demographic encountered in the most recent cohort (mean age 4.68 years compared to approximately 3.3 years in previous periods). These biometric parameters are highly interconnected with age and contribute to the natural process of emmetropization. The continuous documentation of these parameters reinforces their predictive value in preoperative planning, dictating whether an eye can anatomically accommodate a specific IOL type.

Femtosecond laser-assisted cataract surgery (FLACS) was introduced at our institution during the most recent study period and was used in 13 eyes, all of which were operated between 2023 and 2025. This represents an institutional trend rather than evidence of broad applicability and the small number of cases, together with the absence of postoperative outcome data, precludes any conclusion regarding the safety or general feasibility of the technique in pediatric eyes. From an anatomical perspective, the mean WTW diameter exceeded 11.1 mm in all study periods, which is descriptively compatible with the docking requirements of current laser platforms; this observation alone, however, cannot be equated with procedural feasibility. Conceptually, FLACS remains of interest for creating precise anterior and posterior capsulotomies in children, minimizing the risk of radial tears and facilitating advanced techniques such as bag-in-the-lens implantation or posterior optic capture [7,16]. Particularly for early primary IOL implantations, where standard lens designs often struggle to maintain long-term stability, a primary femto-assisted posterior capsulotomy (PPLC) may pave the way for future IOL innovations, such as capsulotomy-fixated lenses that lock directly into the capsule to ensure centration and a permanently clear visual axis [16]. These considerations remain hypothetical and require prospective evaluation. Furthermore, irrespective of the temporal cohort, we maintain that anterior vitrectomy remains an essential adjunct when performing lensectomy in congenital cataracts to prevent VAO and posterior synechiae [17,18].

Our study has several limitations. Its retrospective design and the variability in sample sizes within the surgical subgroups, particularly the ciliary sulcus and iris-fixated groups, limit the strength of subgroup-specific conclusions. Most importantly, the study focuses on preoperative biometry and intraoperative decision-making from a surgeon-centered perspective and does not include postoperative outcomes such as visual acuity, refractive outcome and prediction error, secondary glaucoma, visual axis opacification, IOL decentration or dislocation, reoperation or follow-up duration; these data were not uniformly documented across the entire study period. It therefore cannot be determined from our results whether the observed temporal changes translated into improved patient outcomes. In addition, demographic fluctuations, such as the marked male predominance in the most recent cohort, may incidentally influence overall mean biometric values. A dedicated analysis of postoperative outcomes is planned as a subsequent study.

In conclusion, our longitudinal analysis shows that, while the fundamental biometric criteria guiding IOL placement in pediatric cataracts remain robust, surgical strategies undergo subtle temporal changes. Across the cohort, children were operated on at a progressively older age over the study period, reflecting an ongoing refinement in surgical timing and indication; this trend was particularly apparent within the small ciliary sulcus subgroup, although it was not confirmed as lens-specific in the correlation-adjusted analysis. Preoperative biometrics, particularly axial length and white-to-white diameter, remain the cornerstone of surgical planning, both for conventional surgery and for identifying anatomically suitable candidates for FLACS. Continued monitoring of these temporal trends is essential to further standardize pediatric cataract management and to optimize long-term visual and anatomical outcomes.

## Figures and Tables

**Table 1:** Demographics and baseline clinical characteristics of the study population stratified by study period.

	Group 1 (2019–2020)	Group 2 (2021–2022)	Group 3 (2023–2025)
Total *N* (eyes)	99	90	102
Total *N* (patients)	50	47	51
Traumatic Cataract	3 (3.0%)	0 (0.0%)	5 (4.9%)
Marfan Syndrome	5 (5.1%)	1 (1.1%)	1 (1.0%)
Uveitis	5 (5.1%)	1 (1.1%)	4 (3.9%)
Age at surgery (years)	1.08 [0.11; 15.91]	1.33 [0.11; 15.37]	3.13 [0.14; 15.85]
White-to-White (mm)	11.19 ± 1.19	11.39 ± 1.10	11.46 ± 1.16
Axial-length (mm)	18.91 ± 2.83	19.96 ± 2.42	20.32 ± 3.19
Preoperative IOP	13.15 ± 3.72	14.07 ± 2.32	15.32 ± 2.79
Central corneal thickness (µm)	590.88 ± 53.96	571.00 ± 46.16	597.60 ± 62.04
Kf (mm)	7.72 ± 0.52	7.79 ± 0.51	7.72 ± 0.58
Ks (mm)	7.35 ± 0.55	7.46 ± 0.52	7.36 ± 0.57
Femtosecond-laser asstisted surgery	0	0	13
Male/Female	51/48 (51.5/48.5%)	34/56 (37.8/62.2%)	75/27 (73.5/26.5%)

Data are presented as counts (*n*) and percentages (%) for categorical variables. Age at surgery showed a pronounced right-skewed distribution (Shapiro–Wilk, all *p* < 0.001) and is therefore reported as the median [minimum; maximum]; the biometric parameters were approximately normally distributed and are reported as the mean ± standard deviation (SD). Sex distribution is reported on a per-eye basis. Five patients were operated on their two eyes in different study periods and are each counted once, in the period assigned here; the cohort therefore comprises 291 eyes of 148 unique patients.

**Table 2:** Age at surgery stratified by intraocular lens (IOL) localization and study period.

IOL-Localization	Group	*N*	Median [Min; Max] or Mean ± SD (Years)
aphakia	1 (2019–2020)	43	0.24 [0.11; 7.76]
	2 (2021–2022)	35	0.26 [0.11; 4.01]
	3 (2023–2025)	38	0.38 [0.14; 15.78]
in-the-bag	1 (2019–2020)	22	5.92 [1.38; 15.91]
	2 (2021–2022)	25	7.69 [1.57; 15.37]
	3 (2023–2025)	27	6.70 [1.13; 15.85]
control	1 (2019–2020)	23	1.38 [0.11; 12.08]
	2 (2021–2022)	22	0.85 [0.11; 10.47]
	3 (2023–2025)	27	2.67 [0.14; 15.85]
sulcus	1 (2019–2020)	6	1.49 ± 1.11 [0.47; 3.57]
	2 (2021–2022)	7	3.03 ± 2.97 [0.70; 8.26]
	3 (2023–2025)	8	4.80 ± 3.48 [2.36; 12.73]
IFIOL	1 (2019–2020)	5	11.76 ± 3.65 [8.03; 15.45]
	2 (2021–2022)	1	3.54 [3.54] †
	3 (2023–2025)	2	5.96 ± 0.52 [5.59; 6.33]

Data are presented as the number of eyes (*n*) and the age at surgery in years, given as the mean ± standard deviation (SD) for subgroups with fewer than 10 eyes and as the median for subgroups with 10 or more eyes. The full range (minimum–maximum) is shown in parentheses. This table is descriptive; the statistical comparison of age across the study periods was performed with the mixed-effects model reported in the Methods and Results, which showed a significant overall temporal effect without a significant effect of IOL localization or a localization-by-period interaction. IFIOL = iris-fixated intraocular lens. † SD could not be calculated for this subgroup due to a limited sample size (*n* = 1).

**Table 3:** Anatomical Localization of Implanted IOLs and Aphakia Before and After March 2023.

IOL-Localization	Before 03/2023 (*n* = 153)	After 03/2023 (*n* = 66)	Total (*N* = 219)	*p*-Value
aphakia	85 (55.6%)	31 (47.0%)	116 (53.0%)	0.54
in-the-bag	47 (30.7%)	27 (40.9%)	74 (33.8%)	
sulcus	15 (9.8%)	6 (9.1%)	21 (9.6%)	
IFIOL	6 (3.9%)	2 (3.0%)	8 (3.7%)	

## Data Availability

Data are available upon reasonable request.
